# Early illustrations of the importance of systematic phenotyping

**DOI:** 10.1038/s41431-022-01165-z

**Published:** 2022-10-03

**Authors:** Reuben J. Pengelly

**Affiliations:** Faculty of Medicine, University of Southampton, Southampton, UK

**Keywords:** Genetics research, Genetics

The Online Mendelian Inheritance in Man (OMIM) database (www.omim.org) [1] (and its offline predecessor) has long been a ubiquitous reference for medical genetics professionals and researchers. At its migration from a textbook to an online resource in the 1980s, it was ahead of its time in many ways, though of course the fast-evolving field of medical genetics and genomics now has considerations that would not have been imagined at the time. Notably, the human-readable information in OMIM at the time was not well suited for computational analysis.

To address the challenges of computational analysis of OMIM data at the time, van Driel et al. [2] undertook a text-mining analysis, published in 2006. In this, they extracted MeSH terms present in the text of 5080 OMIM records for disease phenotypes and clustered these records based on similarity in MeSH terms within the records. On exploring the similar OMIM disease phenotypes, they identified that phenotypically linked diseases were more likely to have a shared molecular basis, and furthermore, pathogenic variants in genes in the same family (or containing same functional domains) were more likely to underlie phenotypically similar diseases. Exploring their dataset may support the identification of candidate genes for diseases with unknown molecular bases. The results of their analysis were released in their online tool MimMiner (http://www3.cmbi.umcn.nl/MimMiner), allowing the user to explore an OMIM record in relation to phenotypically similar records.

Since 2006, there has been substantial progress in systematic phenotyping, notably including the development of the Human Phenotype Ontology (HPO) [3]. This ontology has been developed with reference to databases including OMIM. HPO is increasingly being incorporated into routine genomics, and is underpinning the implementation of national genomics programmes such as the 100,000 Genomes Project [4] and the subsequent business as usual NHS Genomic Medicine Service.

Software utilising HPO for diagnostics, such as Exomiser and PhenIX, utilise HPO terms for a patient to identify variants in a patient in genes that would be expected to cause the phenotype from the literature. These software allow for the systematic interpretation of genomic data, streamlining interpretation and allowing efficient use of time for busy clinical staff [5]. Systematic collection of detailed phenotypes when patients are referred for diagnostic testing further allows the leveraging of patient cohorts for research; this allows long-term diagnostic uplift through gene discovery [6].

Phenotyping now with systematic taxonomies in the first instance renders text-mining approaches, such as that applied by van Driel et al. [2] unnecessary, but they are still a useful tool to extract information *post hoc*, allowing valuable information to be pulled into databases going forwards. In the world of ‘big data’, consistency and robustness of data is key. With any data analysis, the results are always limited by the quality of the information going in, so any work we do to improve data quality will pay dividends.
